# Primary sclerosing epithelioid fibrosarcoma of the iliac fossa: a case report

**DOI:** 10.3389/fonc.2026.1823849

**Published:** 2026-05-28

**Authors:** Mengping Li, Zhen Chong, Yuan Jin, Yueqin Chen, Shujun Zhang

**Affiliations:** 1 Department of Radiology, Affiliated Hospital of Jining Medical University, Jining, Shandong, China; 2 College of Medical Imaging and Laboratory, Jining Medical University, Jining, Shandong, China; 3 Department of Pathology, Affiliated Hospital of Jining Medical University, Jining, Shandong, China

**Keywords:** case report, computed tomography, magnetic resonance imaging, retroperitoneal neoplasms, sclerosing epithelioid fibrosarcoma, tumor

## Abstract

**Background:**

Sclerosing epithelioid fibrosarcoma (SEF) is an exceedingly rare and aggressive fibroblastic malignancy. Primary involvement of the retroperitoneal iliac fossa is exceptionally uncommon in the literature, and its nonspecific clinical features and overlapping imaging findings pose significant diagnostic challenges.

**Case presentation:**

This case report describes a 53-year-old man who presented with a large, painful, palpable mass in the right lower quadrant. Imaging revealed a well-circumscribed lesion in the right iliac fossa. Non-contrast-enhanced computed tomography (CT) showed heterogeneous low density, while contrast-enhanced CT demonstrated characteristic progressive enhancement. On magnetic resonance imaging (MRI), the lesion exhibited iso- to slightly hypointense signals on T1-weighted imaging, mixed hyperintensity on T2-weighted imaging, and remarkable heterogeneous restricted diffusion on diffusion-weighted imaging. The patient underwent radical surgical resection, and the diagnosis of SEF was confirmed by histopathological and immunohistochemical examinations. No evidence of recurrence or metastasis was observed during the short-term follow-up period.

**Clinical discussion:**

The uniqueness of this case lies in the primary iliac fossa location, which has been reported only once previously in the pathological literature. Preoperatively, differential diagnoses included solitary fibrous tumor, low-grade fibromyxoid sarcoma, and schwannoma, which were ruled out based on characteristic imaging and histopathological features. During 10 months of short-term follow-up, there was no evidence of local recurrence or distant metastasis.

**Conclusions:**

A deep-seated soft-tissue mass displaying heterogeneous T2 signal with hypointense collagenous areas and progressive enhancement should raise suspicion for sclerosing epithelioid fibrosarcoma, especially in atypical locations such as the iliac fossa. Definitive diagnosis requires histopathology and immunohistochemistry (MUC4), while long-term follow-up is essential to monitor late recurrence or metastasis.

## Introduction

Soft tissue sarcomas (STSs) comprise a heterogeneous group of mesenchymal malignancies, accounting for approximately 1% of all adult cancer cases (1). Sclerosing epithelioid fibrosarcoma (SEF) is an exceptionally rare subtype, which was initially characterized by Meis-Kindblom et al. in 1995 (2). To date, over 100 cases have been reported in the literature, representing less than 1% of all STS cases. While SEF can arise in soft tissues or bone throughout the body, it most frequently affects the soft tissues of the extremities, trunk, and head and neck (3). Primary involvement of the iliac fossa remains exceptionally rare, with only one previously documented case, which lacked comprehensive imaging data (4). This makes our case the second reported instance of iliac fossa SEF and the first to include detailed multimodal imaging. Historically, SEF was considered as a low-grade neoplasm. However, the 2013 World Health Organization (WHO) classification of tumors of soft tissue and bone reclassified SEF as a malignant fibroblastic tumor, reflecting its aggressive potential for local recurrence and late-stage metastasis (5).

Research on SEF has predominantly concentrated on its pathological features and molecular mechanisms, leaving a significant void in systematic imaging analyses, particularly for retroperitoneal cases. Preoperative misdiagnosis remains a challenge because the imaging manifestations of SEF mainly overlap with more common retroperitoneal tumors, such as solitary fibrous tumors (SFTs), low-grade fibromyxoid sarcomas (LGFMSs), and schwannomas. Given that SEF is relatively insensitive to chemotherapy and radiotherapy, radical surgical resection is the primary therapeutic strategy to improve prognosis (6). Consequently, precise preoperative imaging is critical for guiding clinical management and surgical planning.

This report presents a case of primary SEF arising in the iliac fossa and evaluated with multimodal imaging including multidetector CT (MDCT) and magnetic resonance imaging (MRI). Systematic radiologic–pathologic correlation highlights key differential diagnostic features and demonstrates the clinical utility of imaging in improving recognition of this rare entity among radiologists and clinicians.

## Case presentation

A 53-year-old man presented with a mass in the abdomen for 1 year and pain at the site of the mass for 1 day. One year prior to admission, the patient found a quail egg–sized mass in the right lower abdomen that was non-tender and not associated with overlying skin ulceration. No diagnosis or treatment was undertaken at that time, and the mass gradually increased in size. One day before admission, localized tenderness developed at the tumor site without obvious precipitating factors or accompanying symptoms, such as nausea, vomiting, abdominal distension, and diarrhea. Physical examination revealed an irregular, palpable mass in the right lower quadrant, approximately 80mm in diameter. The mass had a rough surface, ill-defined borders, poor mobility, and significant tenderness. Laboratory investigations showed a C-reactive protein (CRP) level of 35.4 mg/L and a monocyte count of 0.77 × 10^9^/L. Both values were at the upper limit of normal, suggesting a possible systemic inflammatory response.

CT imaging (**Figure 1**) demonstrated a large, soft-tissue density mass in the right iliac fossa, which was situated medially to the iliopsoas muscle. The lesion measured approximately 78 × 76 × 92mm, exhibited heterogeneous internal density (mean attenuation: 37 HU), and ill-defined margins relative to the adjacent bowel loops. Non-contrast-enhanced MRI (**Figure 2**) further characterized the retroperitoneal lesion (approximately 77 × 68 × 103mm) as isointense to slightly hypointense on T1-weighted imaging (T1WI) and demonstrating heterogeneous hyperintensity on T2-weighted imaging (T2WI). Compared with CT, the mass had relatively well-defined margins on MRI, with clear displacement of adjacent structures. Diffusion-weighted imaging (DWI) displayed heterogeneous high signal intensity, with corresponding low apparent diffusion coefficient (ADC) values, indicating restricted diffusion. On contrast-enhanced CT (**Figure 3**), the lesion exhibited a characteristic heterogeneous “progressive” enhancement pattern, with attenuation values of approximately 46 HU in the arterial phase, 51 HU in the venous phase, and 56 HU in the delayed phase. Adjacent vessels were compressed, and the arterial supply was inferred to originate from small branches of the superior mesenteric artery and the external iliac artery.

**Figure 1:**
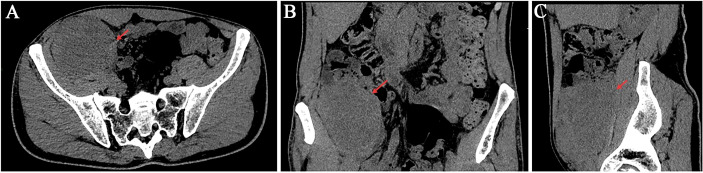
Abdominal computed tomography images. A lobulated mass is observed medial to the right iliopsoas muscle in the iliac fossa [**(A)** axial; **(B)** coronal; **(C)** sagittal views], exhibiting ill-defined margins with the adjacent intestinal loops.

**Figure 2:**
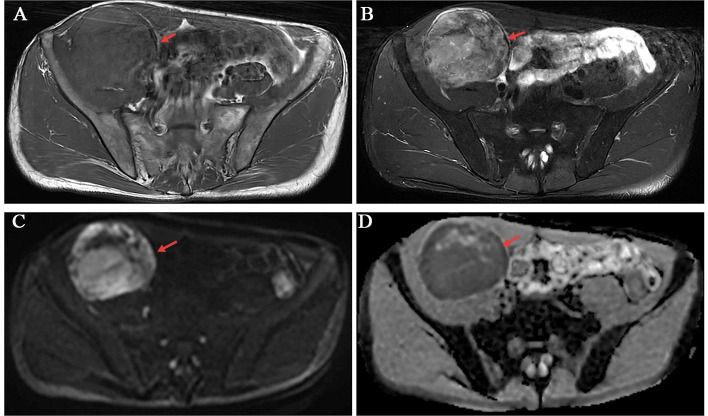
Preoperative MRI findings. **(A)** T1-weighted imaging (T1WI) shows a slightly hypointense signal; **(B)** T2-weighted imaging (T2WI) reveals a heterogeneous, mildly hyperintense signal interspersed with linear hypointensities; **(C, D)** Diffusion-weighted imaging (DWI) and the corresponding apparent diffusion coefficient (ADC) values (mean ADC: 0.91 × 10^-^³ mm²/s) demonstrate heterogeneous hyperintensity and hypointensity, respectively, indicating remarkable diffusion restriction in the mass.

**Figure 3:**
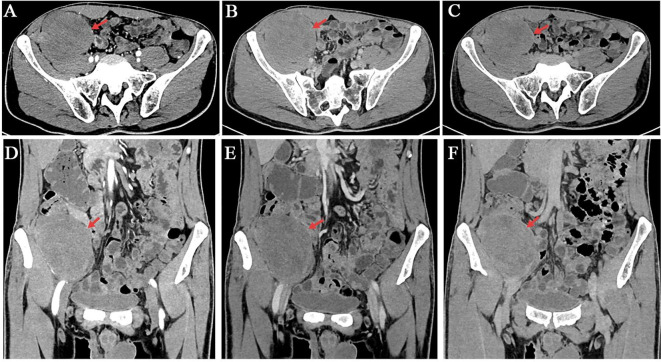
Abdominal contrast-enhanced computed tomography images. [**(A–C)** axial; **(D–F)** coronal; representing arterial, venous, and delayed phases, respectively]. The lesion shows heterogeneous and progressive enhancement, with CT values of approximately 46, 51, and 56 HU across the three phases. Adjacent vessels are compressed, and the blood supply is derived from small branches of the superior mesenteric artery and the external iliac artery.

Histopathological examination (**Figure 4A**) revealed relatively uniform, small-to-medium-sized round or oval tumor cells embedded in stroma characterized by abundant, deeply eosinophilic collagen fibers. Immunohistochemical staining (**Figures 4B–D**) revealed that the tumor cells were positive for MUC4, CD99, and Ki-67 (proliferation index of approximately 5%), with focal positivity for Vimentin; however, the lesion was negative for diverse markers, including CK, S-100, CD34, SS18-SSX, SMA, Desmin, STAT-6, CD117, Dog-1, Myogenin, NKX2.2, and LCA. Based on these morphological features and the definitive immunohistochemical staining, particularly the expression level of MUC4, the lesion was diagnosed as a mesenchymal tumor with associated necrosis, which was confirmed as SEF of the right iliac fossa.

**Figure 4:**
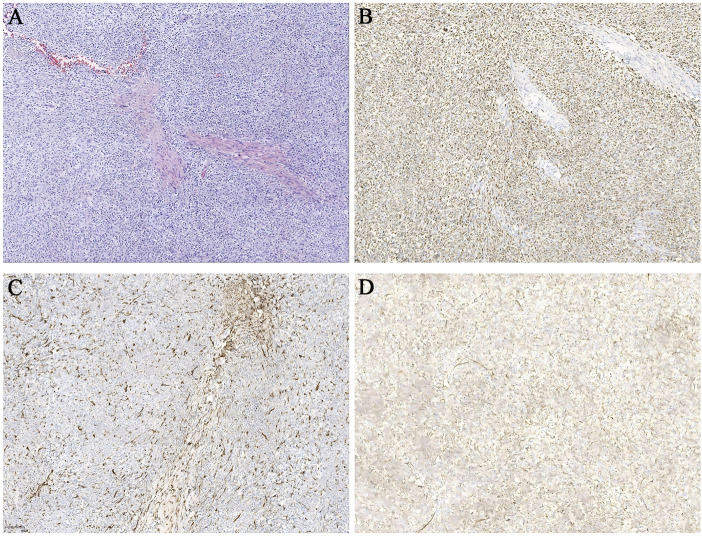
Histopathological and immunohistochemical features. **(A)** Hematoxylin and eosin (H&E) staining (100×) shows relatively uniform tumor cells, primarily small-to-medium-sized, round or oval in shape, with abundant eosinophilic collagen fibers in the stroma; **(B–D)** Immunohistochemical staining (100×) reveals diffuse positivity for MUC4 **(B)**, Vimentin **(C)**, and CD99 **(D)**.

The patient underwent “resection of the retroperitoneal tumor” under general anesthesia combined with a regional nerve block. Intraoperatively, a small amount of serous (light yellow) ascites was observed. The liver surface was smooth, with no palpable nodules. The greater omentum was adhered to the right lower quadrant. The tumor was located in the right iliac fossa, measuring approximately 150 × 130mm. It exhibited ill-defined borders, was fixed in position, and showed significant adhesions to the cecum and ascending colon. A nodular specimen measuring 106 × 80 × 72mm was excised. The cut surface exhibited a variegated appearance with intermixed grayish-white, grayish-yellow, and grayish-brown areas, and the consistency was firm.

Postoperative recovery was uneventful, and the patient was discharged on day 8. Periodic surveillance via pelvic MRI at 1 and 4 months postoperatively revealed no evidence of residual disease or local recurrence in the right iliac fossa. The previously displaced iliac vessels and bowel loops had returned to their normal anatomical positions, and no ascites was detected. Slightly enlarged lymph nodes were identified in the bilateral inguinal regions. During the 10-month short-term follow-up period, the patient remained asymptomatic with no imaging evidence of recurrence or metastasis. Given SEF’s known risk of late metastasis (up to several years post-resection), the patient is undergoing long-term surveillance with annual chest CT.

## Discussion

SEF is a rare and aggressive malignant fibroblastic neoplasm (7). Most existing literature on SEF consists of pathological series or isolated case reports, with limited emphasis on comprehensive radiological characterization, especially for retroperitoneal locations such as the iliac fossa. SEF typically affects middle-aged adults, with a median age of 45 (range: 38–55) years and no significant sex predilection (8, 9); our patient’s profile aligns with these reported demographics. While SEF primarily arises in the deep musculature of the extremities, trunk, and head and neck, visceral involvement (including the kidney, liver, pancreas, and gastrointestinal tract) has been sporadically reported. Notably, primary involvement of the iliac fossa is exceptionally rare; to our knowledge, only one prior case has been documented in the pathological literature, and that report lacked detailed imaging (4). Thus, the present case represents the second reported iliac fossa SEF and the first with comprehensive multimodal CT and MRI characterization. The complex anatomy of the iliac fossa, acting as a transition zone between the abdomen and pelvis, often allows these tumors to remain asymptomatic until they achieve substantial dimensions. This often results in a significant discrepancy between tumor volume and symptom severity at discovery, with median diameters at diagnosis reaching up to 20cm (10). Clinical manifestations, such as the localized and radiating pain found in this case, typically stem from the compression of adjacent neurovascular or visceral structures. Despite its historical description as a “low-grade” entity, recent evidence highlights SEF’s aggressive biological behavior, characterized by high rates of local recurrence (27%), metastasis (80%), and mortality (47%) (11). Given its propensity for late-stage pulmonary and pleural spread, occurring in approximately 50% of cases, long-term surveillance via periodic chest CT is mandatory (12).

Preoperative imaging is essential for delineating tumor extent, vascular involvement, and metastatic status, yet radiological reports of SEF remain limited. Previous case reports have noted that SEF in the pancreas may present as a well-circumscribed mass with internal calcifications and centripetal, heterogeneous enhancement (12), while hepatic involvement often manifests as lobulated masses with irregular enhancement (8). On CT scan, SEF generally appears as a large soft-tissue density mass, ranging from 3.2 to 29.0 (median: 8.2) cm, occasionally featuring scattered calcifications or necrotic cystic components (13). In the present case, the tumor exhibited a “progressive” enhancement pattern, with attenuation values gradually increasing from the venous to the delayed phases, a kinetic profile likely attributable to the high proportion of dense sclerotic collagenous stroma. This compact extracellular matrix may impede contrast medium diffusion and prolong interstitial retention, aligning with the enhancement characteristics of fibro-sclerotic tissues. Given that the lungs are frequently the primary site of metastasis, preoperative chest CT is recommended for all retroperitoneal sarcoma patients to guide surgical planning (10). MRI further elucidated the internal architecture, displaying mixed iso-to-high signal intensity on T2WI interspersed with irregular low-signal zones. These hypointense areas correspond to regions of dense collagen deposition or hyaline sclerosis, while the remarkable restricted diffusion on DWI (high signal intensity with low ADC values) confirms high cellularity and compact cell arrangement. For retroperitoneal tumors, MRI remains the modality of choice for assessing muscular invasion and determining optimal surgical margins.

The radiological appearance of SEF requires careful differentiation from other mesenchymal neoplasms, including solitary fibrous tumor (SFT), low-grade fibromyxoid sarcoma (LGFMS), and schwannoma, which were the main preoperative considerations in this case. SFT typically exhibits a characteristic “map-like” mixed signal on T2WI, while in contrast to the progressive enhancement of SEF, SFT is often hypervascular with rapid “wash-in and wash-out” kinetics (14, 15). LGFMS primarily affects younger patients (median age: 29 years) and often presents as a homogeneously bright mass on T2WI due to its prominent myxoid matrix (16, 17). While LGFMS also shows delayed enhancement, its stroma is typically less dense than that of SEF, and it lacks the characteristic epithelioid morphology (18). While both sclerosing epithelioid fibrosarcoma (SEF) and low-grade fibromyxoid sarcoma (LGFMS) may exhibit MUC4 expression, they are distinguished by key morphological differences. In the present case, the tumor demonstrated characteristic epithelioid cells arranged in nests or cords and embedded within a markedly dense, hyalinized collagenous stroma. In contrast, LGFMS typically displays alternating collagen-rich and myxoid areas arranged in a whorled pattern and lacks prominent epithelioid cytomorphology. These distinct histological features formed the fundamental basis for establishing a diagnosis of SEF over LGFMS. Furthermore, schwannomas mainly exhibit the classic “target sign” on T2WI and robust early enhancement, which were distinctly absent in this case (5). Histologically, SEF is defined by nests or cords of small-to-medium epithelioid cells embedded in a densely sclerotic stroma (19). Immunohistochemically, MUC4 serves as a highly sensitive and specific marker, displaying diffuse and strong cytoplasmic positivity in approximately 80% of cases, whereas epithelial markers, such as cytokeratin (CK) and EMA, are typically negative (16). Molecularly, approximately 75% of SEF cases harbor *EWSR1* gene rearrangements, most commonly with *CREB3L1* as the fusion partner, providing a definitive diagnostic basis for challenging cases (20). While molecular characterization of EWSR1 gene rearrangement is a valuable diagnostic adjunct for SEF, it was not performed in the current case. Nevertheless, the diagnosis was robustly established based on the pathognomonic histopathological features and diffuse MUC4 immunoreactivity, which is a highly sensitive and specific marker for this entity. We acknowledge that the absence of molecular confirmation represents a limitation of this study. Macroscopically, these tumors are often nodular or lobulated with a firm, grayish-white cut surface, occasionally accompanied by cystic change, calcification, or necrosis (16).

The mild elevation of C-reactive protein (CRP) observed in this case may be attributed to local or systemic inflammation secondary to the substantial tumor bulk. While inflammatory markers have prognostic relevance in certain sarcomas, their specific significance in SEF remains unclear and warrants further investigation in larger cohorts (21).

Due to the rarity of SEF, standardized therapeutic protocols have not yet been established. Radical surgical resection remains the primary treatment strategy, as the tumor is notoriously resistant to traditional chemotherapy and radiotherapy (6, 22). While the role of adjuvant therapy is controversial, studies have demonstrated that anthracycline-based regimens have limited efficacy (23), whereas topoisomerase I inhibitors (e.g., irinotecan) or targeted immunotherapies, including ALK inhibitors and immune checkpoint inhibitors, show emerging promise (24–26). Radiotherapy may be prescribed for tumors that are unresectable or have close surgical margins (27). Given the poor 5-year survival rate and the propensity for late pulmonary metastasis, long-term clinical and radiological follow-up is essential (11).

## Conclusion

In conclusion, primary SEF of the iliac fossa represents an exceedingly rare diagnostic entity. While individual imaging features may lack pathognomonic specificity, the combination of a deep-seated soft-tissue mass demonstrating heterogeneous T2-weighted signal intensity, particularly irregular low-signal areas corresponding to a dense collagenous matrix, together with a characteristic progressive enhancement pattern, may provide suggestive but non-specific radiologic features that can assist in raising suspicion for SEF. These imaging findings should raise a strong index of suspicion for sclerotic fibro-collagenous malignancies, such as SEF. For such patients, although no recurrence was observed during short-term follow-up, long-term clinical and radiological surveillance is essential due to the risk of late recurrence and metastasis in SEF. A heightened awareness of these imaging hallmarks, accompanied by a multimodal diagnostic approach, is essential for timely diagnosis, precise surgical mapping, and ultimately, optimizing the therapeutic trajectory for these patients. Given the single-case nature of this report, these findings should be considered hypothesis-generating and warrant further validation.

## Data Availability

The original contributions presented in the study are included in the article/supplementary material. Further inquiries can be directed to the corresponding authors.
